# Use of a German longitudinal prescription database (LRx) in pharmacoepidemiology

**DOI:** 10.3205/000218

**Published:** 2015-08-25

**Authors:** Hartmut Richter, Silvia Dombrowski, Hajo Hamer, Peyman Hadji, Karel Kostev

**Affiliations:** 1 IMS Health, Real World Evidence Solutions, Frankfurt, Germany; 2 Department of Neurology, Epilepsy Center, University of Erlangen, Germany; 3 Department of Women’s Health, North West Hospital, Frankfurt, Germany; 4 Fresenius University of Applied Sciences, Idstein, Germany

**Keywords:** database, LRx, epidemiology

## Abstract

Large epidemiological databases are often used to examine matters pertaining to drug utilization, health services, and drug safety. The major strength of such databases is that they include large sample sizes, which allow precise estimates to be made. The IMS^®^ LRx database has in recent years been used as a data source for epidemiological research. The aim of this paper is to review a number of recent studies published with the aid of this database and compare these with the results of similar studies using independent data published in the literature. In spite of being somewhat limited to studies for which comparative independent results were available, it was possible to include a wide range of possible uses of the LRx database in a variety of therapeutic fields: prevalence/incidence rate determination (diabetes, epilepsy), persistence analyses (diabetes, osteoporosis), use of comedication (diabetes), drug utilization (G-CSF market) and treatment costs (diabetes, G-CSF market). In general, the results of the LRx studies were found to be clearly in line with previously published reports. In some cases, noticeable discrepancies between the LRx results and the literature data were found (e.g. prevalence in epilepsy, persistence in osteoporosis) and these were discussed and possible reasons presented. Overall, it was concluded that the IMS^®^ LRx database forms a suitable database for pharmacoepidemiological studies.

## Zusammenfassung

Große epidemiologische Datenbanken werden oft eingesetzt, um Fragen der Nutzung von Medikamenten, der Versorgungsforschung und der Arzneimittelsicherheit zu untersuchen. Die Hauptstärke solcher Datenbanken ist ihr großer Umfang, der sehr präzise Aussagen gewährleistet. Die Datenbank IMS^®^ LRx ist in den letzten Jahren vermehrt als Datenquelle für epidemiologische Studien eingesetzt worden. Ziel dieser Arbeit ist es, eine Reihe neuerer Studien, die mit Hilfe dieser Datenbank durchgeführt wurden, zu rezensieren und Vergleiche mit den Ergebnissen ähnlicher Studien in der Literatur zu erstellen. Trotz der Beschränkung auf Originalarbeiten, für die vergleichbare unabhängige Studien in der Literatur zu finden waren, war es möglich, eine größere Auswahl von Analysebeispielen in diversen Märkten zu treffen: Ermittlung der Prävalenz/Inzidenz (Diabetes, Epilepsie), Persistenzanalysen (Diabetes, Osteoporose), Komedikation (Diabetes), Medikamentennutzung (G-CSF Markt) und Behandlungskosten (Diabetes, G-CSF Markt). Im allgemeinen stimmten die Ergebnisse der LRx-Studien mit denen der Vergleichsanalysen klar überein. In manchen Fällen gab es jedoch deutliche Abweichungen zwischen den LRx- und den Literaturergebnissen (z.B. Prävalenz der Epilepsie, Persistenz bei Osteoporosebehandlung). Diese wurden diskutiert und mögliche Gründe vorgebracht. Zusammenfassend wurde die IMS^®^ LRx Datenbank als geeignet betrachtet, pharmakoepidemiologische Studien durchzuführen.

## Introduction

Large epidemiological databases are available in many countries. They are often used to examine matters pertaining to drug utilization, health services, and drug safety. The major strength of such databases is that they include large sample sizes, which allow precise estimates [1] to be made. In Germany, health insurance databases are one example of data sources that may be suitable for addressing epidemiological issues [2], [3].

The IMS^®^ LRx database has been used several times as a data source for epidemiological research in recent years. It has access to pharmacy data collection centers nationwide, which process prescription data related to all German patients with statutory health insurance for reimbursement purposes. Data entries comprise patient specific data over time, such as anonymised identification number, age, gender, insurance company, and area of residence as well as prescription information, including prescriber’s anonymised identification number, date and package size [4]. The IMS^®^ LRx database currently contains approximately 60% of all prescriptions reimbursed nationwide.

Studies based on the IMS^®^ LRx database have significant strengths but also limitations. The major strength is the large sample size, which allows precise estimates to be made. Large databases of this kind are usually the only means of investigating prescription frequencies and trends of newly launched products.

The main limitation is the absence of important variables, as the database and the data collection procedures are not designed for specific research investigations. A careful analysis is usually required to determine whether a valid answer to the research question can be achieved on the basis of the data available.

The present work consists of a review of some of the existing studies that have been carried out using IMS^®^ LRx and make comparisons with similar studies from the literature. In choosing the LRx studies, it was attempted to make as broad a selection as possible with regard to markets and types of investigation carried out. However, we were obviously restricted not only to LRx studies already published but also to those for which comparative publications could be found. As such, this review is not a systematic one since no exhaustive search was conducted in order to match all current published LRx studies with at least one independent one. The aim of this paper is now to summarize the investigations carried out using IMS^®^ LRx, to evaluate the comparisons between these and other studies and to discuss possible reasons for any discrepancies found.

## Description of the IMS® LRx database

IMS LRx comprises ca. 60% of all prescriptions reimbursed by statutory health insurance funds in Germany. The discrepancy between this and full coverage is caused by the lack of some pharmacy data collection centres in the database. The most prominent of these is the northern collection centre (NARZ) so that coverage in the north is only ca. 10–20%. The coverage in the south of Germany, on the other hand, approaches 90% and that in the central regions is ca. 50%. In contrast with prescriber databases such as IMS^®^ Disease Analyzer, only claimed/reimbursed prescriptions are recorded. Therefore, the data does not reflect the intention to prescribe a particular product but rather the actual dispensation. There is therefore no problem with unclaimed prescriptions that lead to hypothetical therapy that is actually not carried out. Furthermore, in cases of substitution at product level (generics for originals), only the truly dispensed product is seen, preventing the generation of misleading sales data for particular products. Data is available at patient level whereby all patient information is fully anonymised in accordance with data privacy laws by the data provider. Each prescription is available with full product information (e.g. brand, substance, package size, product form) and prescription information (e.g. number of packages dispensed, dates dispensed/claimed, speciality of prescribing physician). Demographic data of the patient (age, sex, insurance status) is also available. The database lacks further information pertaining to e.g. diagnoses or associated laboratory tests. Set against that, the full history of the patient can be seen across doctor specialities and morbidities (i.e. a patient with e.g. metabolic syndrome is not split into several anonymised patient identities by morbidity).

## Discontinuation of insulin pump therapy

Kostev et al. [5] determined the proportion of pump discontinuation in children, adolescents and young adults using the LRx database. 2,452 patients (age <25 years) with new-onset insulin pump prescriptions between January 2009 and December 2010 that were observable in the database for at least 12 months after their first such prescription were selected. For these patients, subsequent insulin or needle prescriptions were investigated for the observable 12 months after index date. Daily insulin dosages were calculated based on the dates of prescriptions, a prescription being assumed to last until the next such prescription. Discontinuation of insulin pump therapy was defined as a switch to other insulin therapies.

Overall, 177 (7.2%) patients stopped insulin pump treatment within one year. 65.0% of discontinuing patients were female which was significantly higher than those in the overall insulin pump population (55.2%) and the probability of discontinuation was significantly higher in the older age groups than the younger ones (multivariate odds ratios controlling for other parameters: 0.36, 0.90 and 1.30 in age groups 0–6 y, >6–12 y, >12–18 y respectively vs. control group >18–25 y). These results agree with those of other investigations. Hofer et al. [6] recorded a discontinuation rate of 4% among insulin pump patients with girls predominating among the discontinuers in all age groups except the youngest (0–5 y). De Vries et al. [7] noted a higher discontinuation rate of 11.3% over a greater observation time span (up to 5 years). A comparison between discontinuers and 100 randomly selected patients persistent on insulin pump therapy showed that the former had a higher share of female patients (75% vs. 46% in the control) and patients >10 years old (93.8% vs. 80.0% in the control), both comparisons being significant.

14.3% of children with Type 1 diabetes in the study of Kostev et al. [5] received thyroid hormone prescriptions and 2.2% were administered antiepileptics (AED). The shares in both categories increased with age: patients <6 years were treated with thyroid hormone in only 4.0% and AED in only 1.4% of cases but among the 18–<25 year olds, these figures had gone up to 19.6% and 3.4% respectively. In comparison, Kordonouri et al. [8] found a prevalence of more than 15% of thyroid autoimmunity and prescription of thyroid hormones in about 10% of children with type 1 diabetes in 2002. In the study performed by Ramakrishnan and Appleton [9] in the UK, epilepsy occurred in 2.1% of children with type 1 diabetes.

## Treatment with granulocyte colony-stimulating factors (G-CSF)

All G-CSF prescription data between January 2008 and July 2010 from the IMS^®^ LRx database were analyzed to determine G-CSF consumption rates. Based on the individual prescriptions per patient, the total number of G-CSF treatments per patient was determined and the G-CSF number of injections per cycle and total cycle cost was calculated [10]. For those G-CSF preparations administered daily, an average of 4.84–5.42 injections per cycle was calculated. This is in agreement with other studies in which cycle lengths of 5.5 days [11] to 7 days [12] were recorded.

Wasem et al. [13] determined cycle costs for G-CSF preparations based on DDDs as well as on the basis of actual consumption. They demonstrated that filgrastim was theoretically cheaper than lenograstim (cost per cycle based on DDDs: 991.10 Euro for filgrastim, 1,218.20 Euro for lenograstim). This was influenced by the fact that filgrastim was the only G-CSF with biosimilars in the market at time of publication and therefore with cheaper package costs. However, since multiple injections are required for both substances to attain the DDD per dose and since the injection sizes of lenograstim are better suited to achieve this with minimal wastage, the costs per cycle were in actual fact lower for lenograstim (1,359.24 Euro for filgrastim, 1,280.85 Euro for lenograstim). When taking into account that the recommended dose for lenograstim in Germany is distinctly lower than the DDD set by WIdO and that once large injection will suffice here, the cost per cycle for lenograstim drops even further to 807.38 Euro. The third G-CSF product, pegfilgrastim was the most expensive (only one injection required per cycle, total cost 1,632.53 Euro). These trends were also found by Pfannkuche et al. [14] who recorded costs per patient (rather than per cycle) of 1,617.23 Euro for lenograstim, 2,784.18 Euro for filgrastim and 4,345.75 Euro for pegfilgrastim. Both studies confirm the results of Hadji et al. [10], demonstrating that the LRx database records the situation found in everyday life rather than hypothetical scenarios based on recommendations such as DDDs.

## Prevalence and utilization of antiepileptic drugs

Hamer et al. [15] analyzed antiepileptic drug (AED) prescriptions in 2009 based on data from the LRx database. The prevalence was calculated on the basis of data from LRx in comparison with official statistics on the number of patients with statutory health insurance in the population and the use of AED for epilepsy validated using a diagnosis based database (IMS^®^ Disease Analyzer). The study revealed a prevalence of 9.1 per 1,000 of patients taking AED for epilepsy in Germany for 2009. This was similar to a 2007 prevalence of 9.7 per 1,000 in a study using a nationwide Norwegian prescription database [16], the authors of which highlighted the difference between definitions of period and point prevalence. Period prevalences frequently provide higher estimates for the average number of patients taking a certain drug. This also applies to drugs that are usually taken continuously when discontinuation rates are high or compliance is low [15]. This explains why the estimated period prevalence in this study was higher as compared with an average point prevalence of 5.3 per 1,000 for the European population according to a systematic epidemiological review [17].

The study of Hamer et al. [15] also revealed an age-dependent prevalence, with the share of epileptics in the population rising from 5.2 per 1,000 among children and adolescents (<18 y) to 8.9 per 1,000 in the age class 18–64 y, peaking at 12.5 per 1,000 in pensioners (≥65 y). Epilepsy prevalence was also higher in males (9.6 per 1,000) than in the female population (8.4 per 1,000). This is supported by other publications. In a review of epilepsy studies conducted in a number of European countries, Forsgren et al. [18] quoted consistently higher prevalence among men than women in almost all studies. They also noted a higher prevalence in children (range: 3.2 to 5.1 per 1,000; N = 9 studies) than in adults (range: 5.3 to 7.7 per 1,000; N = 4 studies), although when restricted to "active" epilepsy (defined as at least one seizure per patient within the last 5 years), this effect was less clear over the entire range of studies analysed. Hollingworth and Eadie [19] also recorded a slightly higher prevalence in males and noted a rising level of AED use with age. Faught et al. [20] used US data and assessed epilepsy only in the age groups above 65 years. They noted distinct differences in the prevalence between various races, ranging from 5.5 per 1,000 among Asians to 18.7 per 1,000 among Afroamericans. Their prevalence of 10.2 per 1,000 for White Americans is however relatively close to that determined by Hamer et al. [15] for the elderly. Since the German population still consists mainly of people of the Caucasian type, this can be considered a close tally. However, unlike Hamer et al. [15], Faught et al. [20] recorded a nearly equal prevalence among men and women in their study. It is quite possible that this is influenced by the fact that the younger age groups were not included by them but would imply that either the incidence rate is higher in females among the elderly (women "catch up" with respect to prevalence in later years) or there is a greater increase in the death rate among males due to epilepsy than among females (older epileptic men selectively die so that the discrepancy in prevalence erodes). The first effect is contradicted by Faught et al. [20] who recorded almost no difference in incidence among women, the second effect would require further testing.

## Costs of treatment regimens with long-acting insulin

Dippel and Schneider [21] performed a cost comparison between three different long-acting insulin based treatment regimens among the German diabetic population, taking into account concomitant co-prescriptions of other antiglycemic drugs, test strips, lancets and needles using the LRx database as a basis. From the perspective of statutory health insurance (GKV), the annual costs per patient in 2009–2011 for insulin glargine (1,211 Euro) and NPH-insulin (1,224 Euro) regimens were comparable, whereas the insulin detemir regimen was more expensive (1,572 Euro). The overall trend was reflected in the costs for the basal insulins only where NPH was cheapest (glargine: 380 Euro, NPH: 253 Euro, detemir: 448 Euro), as well as the associated bolus insulins (glargine: 305 Euro, NPH: 419 Euro, detemir: 493 Euro) and remaining antidiabetic products (glargine: 526 Euro, NPH: 552 Euro, detemir: 631 Euro).

These findings are in line with comparable analyses based on other sources. Dippel et al. recorded annual costs per patient of 1,338 Euro for glargine [22], [23], 1,374 Euro for NPH [22] and 1,858 Euro for detemir [23] in 2008. These trends are very close, the higher absolute values can probably be explained by the stricter selection process, requiring more frequent insulin administration for the patient to be eligible for the study which would raise annual treatment costs. In a further study using the same stricter selection criteria as well as matched groups to eliminate the effects of other independent parameters, Gölz et al. [24] determined annual costs of 1,428 Euro for glargine, 1,453 Euro for NPH insulin and 1,839 Euro for detemir which could be split into respective costs of 417 (glargine), 302 (NPH) and 500 Euro (detemir) for basal insulin, 360 (glargine), 470 (NPH) and 548 Euro (detemir) for bolus insulin and 651 (glargine), 681 (NPH) and 791 Euro (detemir) for the remaining antidiabetic therapy. These results are also in line with those determined by Dippel and Schneider [21].

## Prevalence of diabetes treatment in Germany

Willert et al. [25] estimated the therapy prevalence of diabetes mellitus in Germany in 2007–2009 based on the LRx database, correcting for areas of low coverage by comparing prescription counts against those in a second database known to have 100% coverage (IMS^®^ Contract Monitor). The main goal of the study was to investigate the validity of the LRx data as a basis for prevalence estimation and the results were determined both nationally and by federal state. Treatment prevalence was found to be 7.7% in Germany overall, which was based on treated diabetes patients (both Type 1 and 2) only. This figure is between those of Rathmann et al. [26] who recorded 7.2% prevalence in 2008–2011 and Köster et al. [27] who determined a prevalence of 8.9% in 2007 and 9.7% in 2009. The noticeable discrepancies can be explained by the different sampling structure: the prevalence of Rathmann et al. was based on diagnosed patients but only included Type 2 diabetics whereas Köster et al. also included only treated patients of both diabetes types but was based on data from only the AOK health insurance in the state of Hesse. Since the AOK is known to have a generally higher share of morbid patients than other statutory health insurances in Germany, this represents a selection bias that would lead to an artificial increase in the prevalence figures.

## Persistence in osteoporosis treatment

Ziller et al. [28] investigated persistence with different treatment regimens available for the treatment of osteoporosis in Germany. The authors described different routes of administration (oral, intravenous, subcutaneous) and dosing and timing intervals. In this study, persistence was defined as the time from treatment initiation to discontinuation or end of the observational period and presented as the proportion of patients who continued receiving their initially prescribed therapy at one year. Discontinuation was defined as a treatment gap of more than 183 days. The highest persistence among all treatment groups was found for intravenous bisphosphonates (BPH) with zoledronic acid having nearly two thirds (65.6%) and ibandronic acid more than half (56.6%) of patients persistent after one year. Parathyroid hormone therapy (PTH), which was mainly made up of teriparatide, also showed high persistence (54.7% overall, 54.3% for teriparatide only). Of the oral BPH, those forms taken on a weekly basis had noticeably higher persistence than forms intended for daily consumption. The difference was more pronounced for alendronic acid (weekly: 44.8% persistence, daily: 17.3% persistence) than for risedronic acid (weekly: 35.2% persistence, daily: 30.3% persistence).

Due to the flexibility with which various parameters in persistence analysis can be set and adjusted (required previous history and treatment-free period for incidence, permitted gap between prescriptions for non-persistence, non-persistence only due to treatment cessation or also due to switching, etc.) it can be difficult to make comparisons with other studies on this subject. Nevertheless, similar analyses were found and these agree reasonably well with the persistence figures of Ziller et al. [28]. In a comparison of oral BPH taken daily vs. weekly in three countries [29], the former group was consistently less persistent (UK: 40%, US: 32%, France: 44%) than the latter group (UK: 52%, US: 44%, France: 51%). Hadji et al. [30] used medical practice rather than pharmacy data to determine a persistence of 42% for oral BPH overall. Since here, a switch from one oral BPH to another did not lead to the patient counting as non-persistent, this value would be expected to be higher than those for the individual BPH quoted by Ziller et al. [28]. However, the permitted gap between prescriptions was only 90 days which counteracted the aforementioned effect. A high degree of persistence for teriparatide (79%) was also found by Ziller et al. [31], the greater persistence than that found by Ziller et al. [28] being influenced by the fact that only patients with severe postmenopausal osteoporosis were selected.

## Limitations and strengths of the LRx database

In general, database analyses limit the interpretation of results depending on the information available. Accordingly, the IMS^®^ LRx database is subject to several limitations. As LRx is a prescription database, it does not contain diagnosis information and all estimations must be based on prescriptions only. In some markets, it is fairly simple to conduct an indication split using only demographic and prescription data (e.g. diabetes Type 1 vs. Type 2), in others, indication splits are practically impossible (e.g. antibiotics market). Another limitation is the absence of data on in-patients as well as phenotypic data such as therapeutic outcomes, co-morbidity and adverse drug reactions. In addition, the database does not contain lab values such as HbA1c and glucose values for diabetes studies or blood pressure information for hypertension studies. The database can therefore not be used for drug safety analyses. The analyses performed using the LRx database are retrospective and do not provide substantial information on factors associated with persistence and compliance as these are not provided by LRx^®^. Therefore, no conclusions can be drawn concerning possible underlying confounders such as population bias, severity of disease, prevalent complications or other individual circumstances. 

Set against this, the LRx database has a large number of strengths. Since the entire prescription history of the patient is available across markets and doctor specialities, therapy flows in defined markets are possible, including the investigation of which speciality is responsible for new introductions, therapy switches, etc. Epidemiological studies including compliance and persistence are possible and made more accurate by the fact that gaps in the therapeutic history of the patients are unlikely. The very high level of coverage (more than half of all statutory prescriptions in Germany) ensures a similarly high degree of statistical confidence in the results obtained in LRx based studies. Analyses in small "orphan drug" markets are made possible only by large coverage which is another distinct advantage of IMS^®^ LRx.

## Conclusion

The results of the LRx studies are generally in line with previously published reports. This is particularly the case when treatment prevalence or incidence are analysed. Comparisons of treatment costs also revealed very similar trends between therapies in LRx vs. comparative studies, although here, the absolute values diverged to a greater degree. It is however not clear whether "insider knowledge" such as differences between list prices and rebated prices for products had been included in generating the comparative results (LRx is based strictly on list prices). The comparisons for persistence gave the greatest deviations between the LRx and comparative results but even here, the overall trends could be emulated with LRx. Persistence analysis is the most sensitive to the exact definitions of the analysis of all the comparative studies considered here and it is possible that these factors were largely to blame for the discrepancies. In summary, we conclude that IMS^®^ LRx forms a suitable basis for pharmacoepidemiological studies.

## Notes

### Competing interests

The authors declare that they have no competing interests.
